# Supplementary material for: Antibiotic collateral sensitivity is contingent on the repeatability of evolution
Source: Nat Commun. 2019 Jan 18;10:334. doi: 10.1038/s41467-018-08098-6 (PMC6338734; doi:10.1038/s41467-018-08098-6)
Supplement: Supplementary file 3 — Description of Additional Supplementary Files [file 41467_2018_8098_MOESM3_ESM.pdf]

Description of Additional Supplementary Files

File Name: Supplementary Data 1
Description: Excel tables containing the triplicate MIC measurements and maximum likelihood estimates for the parental strain and X1-X60 to each of the drugs listed in Supplementary Table 1. P-values for the log-rank test for change from parental senstivity are reported. The values in the tables are not corrected for multiple hypothesis testing. The triplicate measurement of the MICs for X1-X12 at passages 2, 4, 6, 8 and 10 are also presented.

File Name: Supplementary Data 2
Description: Comparison of MICs with %chromosomally aligning reads for X1- X12.
