# Supplementary material for: Antibiotic collateral sensitivity is contingent on the repeatability of evolution
Source: Nat Commun. 2019 Jan 18;10:334. doi: 10.1038/s41467-018-08098-6 (PMC6338734; doi:10.1038/s41467-018-08098-6)
Supplement: Supplementary file 6 — Reporting Summary [file 41467_2018_8098_MOESM6_ESM.pdf]

natureresearch

Corresponding author(s): Jacob G Scott

Reporting Summary

Nature Research wishes to improve the reproducibility of the work that we publish. This form provides structure for consistency and transparency in reporting. For further information on Nature Research policies, see Authors & Referees and the Editorial Policy Checklist.

Statistical parameters

When statistical analyses are reported, confirm that the following items are present in the relevant location (e.g. figure legend, table legend, main text, or Methods section).

n/a	Confirmed	
☐	☒	The exact sample size (*n*) for each experimental group/condition, given as a discrete number and unit of measurement
☐	☒	An indication of whether measurements were taken from distinct samples or whether the same sample was measured repeatedly
☐	☒	The statistical test(s) used AND whether they are one- or two-sided<br>*Only common tests should be described solely by name; describe more complex techniques in the Methods section.*
☒	☐	A description of all covariates tested
☐	☒	A description of any assumptions or corrections, such as tests of normality and adjustment for multiple comparisons
☐	☒	A full description of the statistics including central tendency (e.g. means) or other basic estimates (e.g. regression coefficient) AND variation (e.g. standard deviation) or associated estimates of uncertainty (e.g. confidence intervals)
☐	☒	For null hypothesis testing, the test statistic (e.g. *F*, *t*, *r*) with confidence intervals, effect sizes, degrees of freedom and *P* value noted<br>*Give P values as exact values whenever suitable.*
☒	☐	For Bayesian analysis, information on the choice of priors and Markov chain Monte Carlo settings
☒	☐	For hierarchical and complex designs, identification of the appropriate level for tests and full reporting of outcomes
☒	☐	Estimates of effect sizes (e.g. Cohen's *d*, Pearson's *r*), indicating how they were calculated
☐	☒	Clearly defined error bars<br>*State explicitly what error bars represent (e.g. SD, SE, CI)*

Our web collection on statistics for biologists may be useful.

Software and code

Policy information about availability of computer code

Data collection	We used Masterpure complete DNA purification kits to prep DNA. NexteraXT linbraries were used to prepare the samples further before sequencing on an Illumina NextSeq 500.
Data analysis	We wrote custom software to perform the reported likelihood ratio test. This test is fully described in the methods. The software to perfrom the test as well as the implementation of the mathematical model is available at: TODO<br><br>DNA reads were assembled with velvet. SNVs were called using GATK. Deletions using BLAST searches to DH10B reference sequence and contigs. IS elements were identified using ISfinder and ISseeker.

For manuscripts utilizing custom algorithms or software that are central to the research but not yet described in published literature, software must be made available to editors/reviewers upon request. We strongly encourage code deposition in a community repository (e.g. GitHub). See the Nature Research guidelines for submitting code & software for further information.

Data

Policy information about availability of data

All manuscripts must include a data availability statement. This statement should provide the following information, where applicable:

- Accession codes, unique identifiers, or web links for publicly available datasets
- A list of figures that have associated raw data
- A description of any restrictions on data availability

All MIC data is provided in Supplementary Table 2. All genomic data was uploaded to the NCBI Short read archive (accession code: TODO). The data set used to parametrize the mathematical model was published in Mira et al. 2015.

Field-specific reporting

Please select the best fit for your research. If you are not sure, read the appropriate sections before making your selection.

☐ Life sciences ☐ Behavioural & social sciences ☒ Ecological, evolutionary & environmental sciences

For a reference copy of the document with all sections, see nature.com/authors/policies/ReportingSummary-flat.pdf

Ecological, evolutionary & environmental sciences study design

All studies must disclose on these points even when the disclosure is negative.

Study description	We performed mathematical modeling and high throughput experimental evolution to understand the stochasticity of evolution of E. coli under selection and the consequences in terms of collateral sensitivity.
Research sample	E. coli DH10B
Sampling strategy	We performed as many replicates as feasible and tested for significance using multiple hypthesis corrections
Data collection	Data were collected as triplicate measurement of minimum inhibitory concentration (MIC) through a standardized method (outlined in the Methods). Data collection was performed by J Rutter
Timing and spatial scale	MIC measurements and sanger sequencing were performed daily for 12 replicates (as described). For the remaining 48, these measurements were made only at the end of the 10 day period of evolution.
Data exclusions	Following from the concerns of the reviewers, we re-performed the derivations of the MICs in triplicate. The original data were not used.
Reproducibility	We performed 60 replicates of experimental evolution. However, one key result of the paper is that there is a degree of irreproducibility is inherent in the evolutionary process.
Randomization	N/A
Blinding	N/A

Did the study involve field work? ☐ Yes ☒ No

Reporting for specific materials, systems and methods

Materials & experimental systems

n/a	Involved in the study
☒	☐ Unique biological materials
☒	☐ Antibodies
☒	☐ Eukaryotic cell lines
☒	☐ Palaeontology
☒	☐ Animals and other organisms
☒	☐ Human research participants

Methods

n/a	Involved in the study
☒	☐ ChIP-seq
☒	☐ Flow cytometry
☒	☐ MRI-based neuroimaging
